# Identification of a Novel Salt Tolerance-Related Locus in Wild Soybean (*Glycine soja* Sieb. & Zucc.)

**DOI:** 10.3389/fpls.2021.791175

**Published:** 2021-11-18

**Authors:** Xiaoyang Guo, Jinghan Jiang, Ying Liu, Lili Yu, Ruzhen Chang, Rongxia Guan, Lijuan Qiu

**Affiliations:** The National Key Facility for Crop Gene Resources and Genetic Improvement (NFCRI), Institute of Crop Sciences, Chinese Academy of Agricultural Sciences, Beijing, China

**Keywords:** salinity stress, soybean, gene mapping, *Glycine max*, *Glycine soja*

## Abstract

Salinity is an important abiotic stress factor that affects growth and yield of soybean. NY36-87 is a wild soybean germplasm with high salt tolerance. In this study, two F_2:3_ mapping populations derived from NY36-87 and two salt-sensitive soybean cultivars, Zhonghuang39 and Peking, were used to map salt tolerance-related genes. The two populations segregated as 1 (tolerant):2 (heterozygous):1 (sensitive), indicating a Mendelian segregation model. Using simple sequence repeat (SSR) markers together with the bulked segregant analysis (BSA) mapping strategy, we mapped a salt tolerance locus on chromosome 03 in F_2:3_ population Zhonghuang39×NY36-87 to a 98-kb interval, in which the known gene *GmSALT3* co-segregated with the salt tolerance locus. In the F_2:3_ population of Peking×NY36-87, the dominant salt tolerance-associated gene was detected and mapped on chromosome 18. We named this gene *GmSALT18* and fine mapped it to a 241-kb region. Time course analysis and a grafting experiment confirmed that Peking accumulated more Na^+^ in the shoot *via* a root-based mechanism. These findings reveal that the tolerant wild soybean line NY36-87 contains salt tolerance-related genes *GmSALT3* and *GmSALT18*, providing genetic material and a novel locus for breeding salt-tolerant soybean.

## Introduction

Soil salinization due to excessive application of agrochemicals and poor irrigation practices seriously reduces crop yield, and is an increasing threat to the sustainable development of the world food supply (Munns and Tester, 2008; Zhu, 2016). Soybean, which is rich in oil and protein, is not only used as food but also as an industrial raw material for animal feed. As a moderately salt-tolerant crop plant, soybean yield can be reduced by 40% under salt stress (Papiernik et al., 2005). Salt stress can inhibit soybean seed germination and seedling growth. It is therefore necessary to identify salt tolerance loci for future molecular breeding of salt-tolerant soybean.

Soybean germplasm vary in salt tolerance, which provides an opportunity to study the genetic control of this trait. Early research showed that F_2_ populations of chloride includer×excluder varieties segregated in ratios of three non-necrotic plants (low chloride content) to one necrotic plant (high chloride content), indicating a single gene governs the inheritance of salt tolerance. The gene symbol *Ncl* was proposed for the dominant Cl^−^ excluder allele in soybean (Abel, 1969). Decades later, a codominant random amplified polymorphic DNA (RAPD) marker was reported to be tightly linked with the salt-tolerance gene (Guo et al., 2000). A great deal of research has focused on identifying salt tolerance quantitative trait loci (QTLs)/genes in recent decades. The major salt tolerance QTL was first mapped on linkage group N (LG N) in a segregating population of S-100 (salt tolerant)×Tokyo (salt sensitive; Lee et al., 2004). The salt tolerance locus on LG N was proven to be conserved in both cultivated and wild soybean (Lee et al., 2004; Hamwieh and Xu, 2008; Tuyen et al., 2010; Hamwieh et al., 2011; Ha et al., 2013; Guan et al., 2014a). The candidate gene *Glyma03g32900* (Wm82.a1.v1.1) associated with soybean salt tolerance in this interval was identified by resequencing (*GmCHX1*) and map-based cloning (*GmSALT3*; Guan et al., 2014a; Qi et al., 2014), and was also designated as the chloride excluder locus *Ncl* (Do et al., 2016). *GmSALT3* encodes an endoplasmic reticulum-localized protein in the Cation/Proton Antiporter (CPA2) family of transporters, and is dominantly expressed in root phloem and xylem (Guan et al., 2014b). Through a combination of physiological and genetic approaches, we demonstrated that GmSALT3 promoted soybean salt tolerance *via* restricting Na^+^ loading and conducting Cl^−^ retranslocation from the shoot, thus maintaining high salt tolerance under saline conditions (Liu et al., 2016; Qu et al., 2021). Sequence insertion and deletion cause premature stop codon in some *GmSALT3* alleles, and amino acid changes or variation at an intron splice donor site in other alleles contribute to functional loss of GmSALT3 (Guan et al., 2014b). Other structural variation or coding sequence changes have been observed in diverse soybean germplasm, especially wild soybeans, and kompetitive allele specific PCR (KASP) and PCR-based markers have been developed to screen salt-tolerant soybean accessions (Patil et al., 2016; Lee et al., 2018). Sequencing, SNP-based KASP, and PCR-based electrophoresis assays show a high prediction rate (>95%) in identification of salt-tolerant genotypes, indicating that *Glyma03g32900* is necessary for salt tolerance at the seedling stage (Guan et al., 2014a; Do et al., 2016; Lee et al., 2018). However, we found that a Chinese soybean landrace, Peking, contained a salt-tolerant allele of *GmSALT3* but was salt sensitive (Guan et al., 2014b). The result was confirmed by Patil et al. (2016), who also found another nine lines that did not show the expected correlation between haplotype and phenotype. This raises the possibility that other genes or modifiers may affect salt tolerance in Peking. Salt tolerance QTLs in soybean located on several chromosomes other than Chr. 03 have been identified through genetic analysis and genome-wide association studies (GWAS; Chen et al., 2008; Zeng et al., 2017b). Compared to wild soybean, the genetic diversity of cultivated soybean decreased dramatically due to bottlenecks during domestication and human selection (Lam et al., 2010). Recently, a salt tolerance-related gene, *GsERD15B*, was cloned from a panel of 182 wild soybean lines using GWAS, and a 7-bp insertion in the promoter region appeared to be the functional polymorphism (Jin et al., 2021). An inositol polyphosphate 5-phosphatase gene (*Gs5PTase8*) from wild soybean was shown to increase salt tolerance in plants (Jia et al., 2020). Therefore, wild soybean germplasm are valuable resources for mining novel salt tolerance genes. We here generated two segregating F_2:3_ populations using salt-tolerant wild soybean NY36-87 as the male parent and two salt-sensitive cultivars (Zhonghuang39 and Peking) as the female parents. This was intended to (1) identify salt tolerance loci/genes in NY36-87, and (2) explore the genetic factors underlying salt sensitivity in the soybean landrace Peking, which contains a tolerant haplotype of *GmSALT3*.

## Materials and Methods

### Plant Materials and Growth Condition

The wild soybean line NY36-87 and cultivated soybean germplasm were obtained from the Chinese Academy of Agricultural Sciences (CAAS). Crosses were made between salt-tolerant genotype NY36-87 and two salt-sensitive genotypes: commercial soybean cultivar Zhonghuang39 and soybean landrace Peking. Peking is a salt-sensitive soybean accession with a tolerant haplotype of *GmSALT3* (Guan et al., 2014b; Patil et al., 2016). The two segregating populations, Zhonghuang39×NY36-87 and Peking×NY36-87, contained 649 and 1,022 F_2_ plants, respectively.

### Evaluation of Salt Tolerance

**Figure 1:**
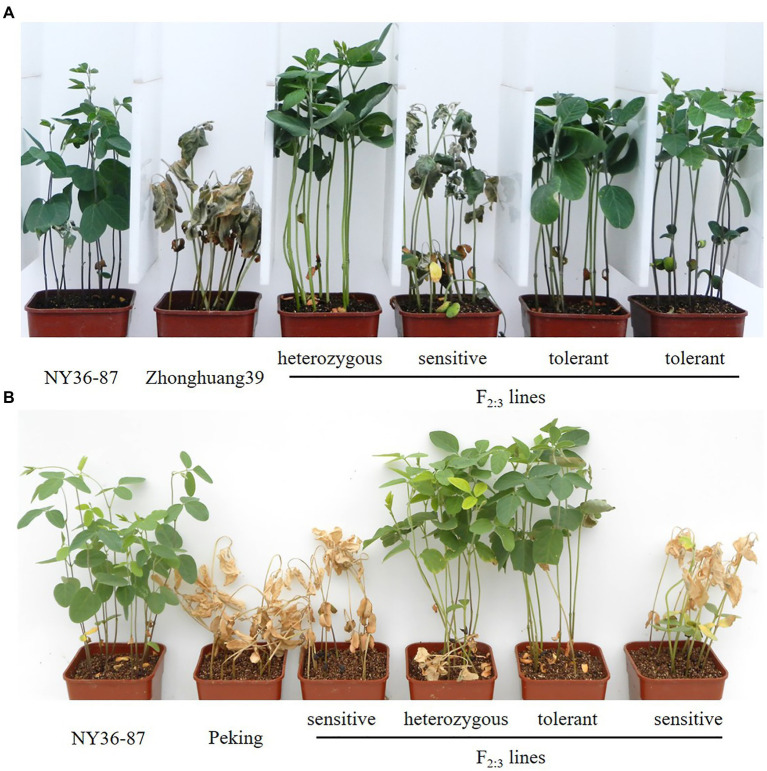
Evaluation of salt tolerance based on leaf scorching in two F_2:3_ populations. **(A)** Parents NY36-87, Zhonghuang39, and example F_2:3_ families. **(B)** Parents NY36-87, Peking, and example F_2:3_ families.

The experiment was conducted in a rain shelter under ambient light and temperature conditions at the Chinese Academy of Agricultural Sciences as previously described (Liu et al., 2016). In brief, for each line, 12 seeds were sown in a 6×6×8cm pot filled with vermiculite. The experiment was conducted in duplicate, with each pot considered a replicate. When unifoliate leaves were fully expanded at 10days after sowing (DAS), pots were subirrigated with 200mM NaCl salt solution once every 2days over the course of 6days. Salt tolerance for each line was assessed based on leaf scorching at 26 DAS. Seedlings in the same pot (families) with normal leaves and no chlorosis were recorded as salt-tolerant; families with all dead plants were recorded as sensitive; families with a mixture of dead (2–5 individuals) and normal (more than five individuals) plants were recorded as heterozygous. In the phenotypic evaluation, 182 families from the cross of Zhonghuang39×NY36-87 and 220 families of Peking×NY36-87 were used (Figure 1).

Soybean plants used for gene expression analysis and grafting experiments were grown in a growth chamber with a 16h light (28°C)/8h dark (25°C) cycle at 60% humidity.

### Na^+^, K^+^ Content Measurement and Grafting

Seeds of salt-tolerant cultivar Tiefeng 8 and salt-sensitive landrace Peking were planted in 6×6×8cm pots containing vermiculite. When unifoliate leaves were fully expanded at 10 DAS, pots were subirrigated with 200mM NaCl salt solution once every 2days over the course of 4days. The roots, stems, and leaves of each pot were harvested separately at 0, 1, 3, 5, and 7days after salt treatment, Na^+^ and K^+^ contents were measured as described by Liu et al. (2016).

For grafting experiment, plants were thinned to five per pot at five DAS and used for grafting, and one pot containing five plants was considered one replicate. Shoots were cut 1.5cm below the cotyledons, and the hypocotyl of the stock was split vertically to a depth of 1.5cm. The base of each shoot scion with cotyledons was cut to form a “V” shape. The scion was inserted into the split rootstock and wrapped with Parafilm. Salt treatment was applied from 8days after grafting. The roots, stems, and leaves of each pot were harvested separately after 10days of salt treatment, and Na^+^ content was measured.

### DNA Extraction and Genotyping

DNA was extracted from leaf samples of each F_2_ individual using the Genomic DNA Purification Kit (Thermo Scientific, United States). Amplification was in a 20μl reaction system, including 1×PCR buffer (TransGen Biotech, Beijing, China), 60ng DNA template, 10pmol of forward and reverse primers, 0.25mM dNTPs, and 1U Easy*Taq* polymerase (TransGen Biotech). The thermocycler program was as follows: 94°C for 5min; 34cycles of 94°C for 30s, 55°C for 30s, and 72°C for 40s; and 72°C for 5min. The PCR products were separated on a 1.5% agarose gel (InDel marker) or 6% denaturing polyacrylamide gel (SSR markers).

### Data Analysis and Gene Mapping

Phenotype of each F_2:3_ family was recorded as tolerant, heterozygous or sensitive in the two segregating populations. The fitness of 1:2:1 (tolerant: heterozygous: sensitive) segregation in the two populations was analyzed using Chi square analysis. Differences of ions accumulation and gene express between Peking and salt tolerant soybean accession were compared using SPSS 18.0 (SPSS Inc., Chicago, IL).

Bulk segregant analysis (BSA) was performed to identify the SSR markers linked to salt tolerance loci in each population. According to the phenotype classification for each F_2:3_ family, 20 homozygous salt-tolerant and 20 homozygous salt-sensitive F_2_ plants were bulked to represent the salt-tolerant pool and salt-sensitive pool. A total of 543 pairs of SSR primers (Supplementary Figure 1) from the soybean genome were used to amplify the DNA of parents and bulked DNA. Gene mapping was performed using QTL IciMapping software version 4.2 (Zhang et al., 2012).^1^ Primers used for gene mapping were listed in Supplementary Table 1.

### Quantitative Real-Time PCR

Total RNA was extracted from root tissues of NY36-87 and Peking using TRNzol Universal Reagent (TransGen Bioteck, Beijing, China). First-strand cDNA synthesis was performed with PrimeScript RT Reagent Kit (TaKaRa, Japan) and 2μg RNA per sample. Quantitative real-time PCR (qRT-PCR) was performed using PerfectStart Green qPCR SuperMix (Tiangen Biotech, Beijing, China). *GmSALT3* expression was calculated using the 2^-ΔΔCt^ method with *GmUKN1* used as the control gene (Livak and Schmittgen, 2001; Guan et al., 2014b). Primers were listed in Supplementary Table 1.

## Results

### Phenotypic Variation and Salt Tolerance Gene Mapping in Zhonghuang39×NY36-87 Population

**Figure 2:**
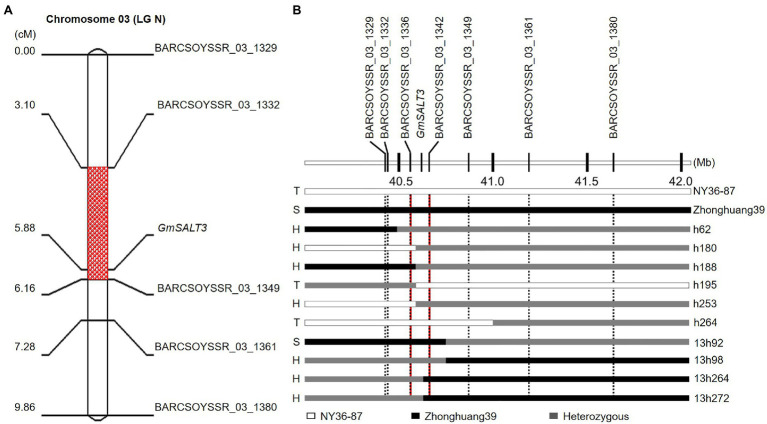
Mapping the salt tolerance locus in the Zhonghuang39×NY36-87 population. **(A)** Salt tolerance-related locus mapped on soybean Chr. 03. **(B)** Fine mapping of the salt-tolerance gene. Individuals are shown to the right and phenotype to the left of the graphic. T, S, and H indicate tolerant, sensitive, and heterozygous (segregating) phenotype, respectively.

F_2:3_ lines of the Zhonghuang39×NY36-87 population were characterized for salt tolerance with 200mM NaCl solution treatment. Salt tolerant, heterozygous, and sensitive lines segregated at 44:93:45, fitting a 1:2:1 ratio (*χ*^2^=0.10<*χ*^2^_(2) 0.05_=5.99; Table 1; Figure 1A). In heterozygous F_2:3_ families, 63% (645 of 1,024) of the F_3_ plants were classified as salt-tolerant, indicating the dominant inheritance of the salt tolerance locus. Because the salt tolerance gene *GmSALT3* located on Chr. 03 has been cloned and confirmed in wild and cultivated soybean germplasm (Guan et al., 2014b), 64 SSR markers from Chr. 03 were primarily used to amplify DNA of parental plants and two bulks. Five polymorphic markers were found between the salt tolerant and sensitive bulks from Zhonghuang39×NY36-87. The 182 F_2_ plants were genotyped using the five polymorphic markers, and the salt tolerance gene was mapped between SSR marker BARCSOYSSR_03_1332 and BARCSOYSSR_03_1349 (Figure 2A). These two markers were used to screen the remaining 631 F_2_ plants of the same population, and 31 recombinants were identified. We screened the salt tolerance in these recombinant lines, and the salt tolerance-related gene was mapped in a narrow interval (98-kb) flanked by BARCSOYSSR_03_1336 and BARCSOYSSR_03_1342, which includes the tolerance gene *GmSALT3*. And *GmSALT3* is co-segregated with the salt tolerance locus on Chr. 03 (Figure 2B). Therefore, we speculate that the dominant gene in NY36-87 may be *GmSALT3*.

**Table 1:** Segregation ratio in F_2_ populations for salt tolerance based on phenotypic evaluation of F_3_ of the crosses made between wild soybean (NY36-87) and two salt-sensitive cultivars (Zhonghuang39 and Peking).

	Zhonghuang39×NY36-87	Peking×NY36-87
Tolerant	44 (45.5)	59 (55)
Heterozygous	93 (91)	105 (110)
Sensitive	45 (45.5)	56 (55)
Expected ratio	1: 2: 1	1: 2: 1
Chi-square (*χ*^2^)	0.10	0.54
*p*-value	0.95	0.72

Expected numbers of F_2_ plants are shown in parentheses.

### Phenotypic Variation and Salt Tolerance Gene Mapping in Population of Peking×NY36-87

**Figure 3:**
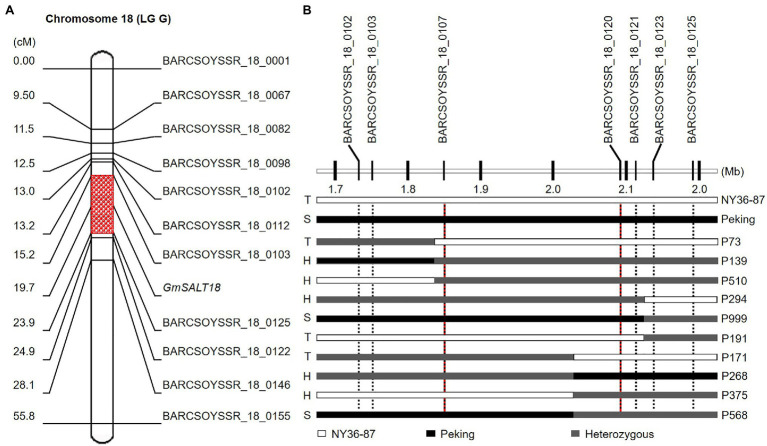
Mapping the salt tolerance locus in the Peking×NY36-87 population. **(A)** Salt tolerance-related locus mapped on soybean Chr. 18 using simple sequence repeat (SSR) markers. **(B)** Fine mapping of salt-tolerance gene. T, S, and H indicate tolerant, sensitive, and heterozygous (segregating) phenotype, respectively.

F_1_ plants of Peking×NY36-87 showed salt tolerance as that of NY36-87. Salt tolerance phenotypic variation in the F_2_ population derived from the cross between NY36-87 and Peking confirmed monogenic inheritance of the salt tolerance phenotype. Among F_2_ individuals, 59 were classified as salt tolerant, 105 as heterozygous, and 56 as salt sensitive according to the phenotype of F_2:3_ lines, indicating that salt tolerance in NY36-87 is controlled by a dominant single gene pair (Table 1; Figure 1B). No polymorphic markers between the salt tolerant and sensitive bulks from Peking×NY36-87 were identified using the SSR markers from Chr. 03, indicating that a new locus other than *GmSALT3* controls salt tolerance in this genetic background. The two DNA bulks from Peking×NY36-87 were screened using 543 SSR markers distributed on the 20 soybean chromosomes. Eleven polymorphic SSR markers from Chr. 18 (LG G) were identified and used to map the salt tolerance gene. This salt tolerance gene on Chr. 18 (which we designated as *GmSALT18*) was mapped to a 430-kb region between BARCSOYSSR_18_0103 and BARCSOYSSR_18_0125 by screening the 220 F_2_ individuals (Figure 3A). To further narrow the preliminary mapping region, BARCSOYSSR_18_0103 and BARCSOYSSR_18_0125 were used to test the remaining 802 F_2_ individuals. In total, 25 recombinants were identified, and *GmSALT18* was fine mapped to a 241-kb interval between BARCSOYSSR_18_0107 and BARCSOYSSR_18_0120 (Figure 3B).

### Peking Accumulated More Na^+^ in Shoots Than Salt Tolerant Soybean Germplasm

**Figure 4:**
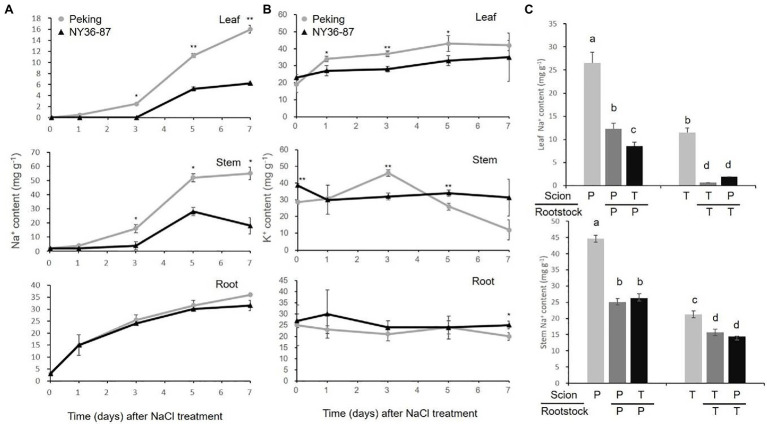
Na^+^ accumulation in Peking and salt-tolerant germplasm. **(A)** The Na^+^ content in Peking (gray) and NY36-87 (black) under 200mM NaCl stress for 0, 1, 3, 5, and 7days. **(B)** The K^+^ content in Peking (gray) and NY36-87 (black) under 200mM NaCl stress for 0, 1, 3, 5, and 7days. **(C)** The Na^+^ content in leaf and stem of non-grafted and grafted soybean Peking (P) and Tiefeng 8 (T) after 10days of salt stress. Letters indicate significant differences between samples. Data shown are the mean of three replicates±SE. ^*^p<0.05, and ^**^p<0.01. Different letters indicate statistically significant differences of ion content in different organs (one-way ANOVA using Fisher’s LSD test, p < 0.05)

Peking accumulated more Na^+^ in shoots compared with salt tolerant soybean cultivars (Liu et al., 2011). Here, to investigate the ion content in Peking and NY36-87, we studied Na^+^ and K^+^ accumulation in roots, stems, and leaves of Peking and NY36-87 over time. Na^+^ content in roots increased during the salt stress, while no significant difference was observed in roots of Peking and NY36-87. Peking accumulated significantly more Na^+^ than NY36-87 in stems and leaves after 5days of salt stress (Figure 4A). No significant difference of K^+^ content in roots of Peking and NY36-87 were observed except for the 7days time point after NaCl treatment, while Peking accumulated more K^+^ in leaves than NY36-87 (Figure 4B). To address the contribution of the root to Na^+^ accumulation in the shoot, we performed a reciprocal grafting experiment between Peking and a tolerant soybean cultivar. Because wild soybean NY36-87 has a slim stem that is difficult to graft with Peking, soybean cultivar Tiefeng 8 was selected as the salt-tolerant germplasm in the reciprocal grafting experiment. The Na^+^ content in leaves and stems of non-grafted Peking (P) and self-grafted Peking (P/P) was much higher than that of non-grafted Tiefeng 8 (T) and self-grafted Tiefeng 8 (T/T; Figure 4C). The Na^+^ content in stem and leaf increased 0.68–13.0-fold in Tiefeng 8 scions when grafted on Peking rootstocks (T/P) compared to self-grafted plants (T/T). These results suggest that Na^+^ accumulation in the shoot of Peking is likely controlled by the rootstock.

### Variation and Expression of *GmSALT3* in Parental Lines

**Figure 5:**
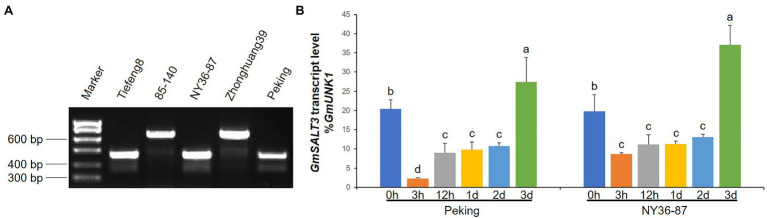
Evaluation of *GmSALT3* in parental lines. **(A)** Genotype confirmation of *GmSALT3* in parental lines using gene-specific marker H2-Insl, salt-tolerant cultivar Tiefeng 8, and salt-sensitive cultivar 85–140 as control accessions. **(B)** Expression of *GmSALT3* in roots of NY36-87 and Peking at different time points after 200mM NaCl treatment. *GmUKN1* was used as the internal reference gene. Letters indicate significant differences between samples (Dunan’s multiple range test at *p*<0.05). Different letters indicate statistically significant differences of gene expression (one-way ANOVA using Fisher’s LSD test, p<0.05)

To validate the variation of the three parental lines, a previously developed InDel marker of *GmSALT3* was used to test DNA of the three parents, using Tiefeng 8 and 85–140 as the salt-tolerant and salt-sensitive control, respectively. The wild soybean NY36-87 and cultivar Peking contain the haplotype H1, as does Tiefeng 8; cultivar Zhonghuang39, like salt-sensitive cultivar 85–140, has the H2 haplotype (Figure 5A). The H2 haplotype is a truncated transcript of *GmSALT3* due to a 3.78-kb retrotransposon insertion in exon 3 (Guan et al., 2014b). To clarify whether salt tolerance in Peking was due to regulation of *GmSALT3*, we quantified *GmSALT3* expression in the roots of NY36-87 and Peking under salt stress. *GmSALT3* showed a similar expression pattern in the roots of NY36-87 and Peking. The transcript abundance of *GmSALT3*, decreased dramatically after 3h of NaCl solution treatment, and recovered to a higher level after 3days (Figure 5B). These results indicated that the salt sensitivity in Peking may not be the result of *GmSALT3* expression alteration.

## Discussion

Over the past half century, scientists have focused on genetic analysis and begun to identify genes that control different traits associated with salt tolerance (Abel and Mackenzie, 1964; Abel, 1969; Shao et al., 1994). Because the traits used in different studies have varied, most salt tolerance gene mapping was performed as QTL using bi-parental populations, natural populations, or a combination of both. Leaf chloride content was considered to be an indicator of soybean response to salinity, and high chloride toxicity to be related to leaf necrosis (Abel and Mackenzie, 1964). Thus, leaf chloride accumulation and necrosis were used to study the genetic factors of salt tolerance in soybean, and the two traits were controlled by a single dominant gene in different genetic backgrounds (Abel, 1969; Shao et al., 1994). Using the salt tolerance rating score that classifies based on leaf scorching symptoms, a major QTL that could explain most of the phenotype variation in greenhouse and field environments was mapped on soybean LG N (Chr. 03; Lee et al., 2004). Nine QTLs were identified in a recombinant inbred line (RIL) population when the traits measured were tolerance rating, survival time, and survival ratio. The locus qppsN.1, tightly linked with Satt237, was located in the same region as the one mapped on LG N (Chen et al., 2008). The major QTL on Chr. 03 (LG N) was independently proven to be a conserved locus (Hamwieh and Xu, 2008; Hamwieh et al., 2011; Ha et al., 2013; Guan et al., 2014a). These studies led to cloning of the candidate gene, *Glyma03g32900*, under this dominant locus (Qi et al., 2014; Guan et al., 2014b; Do et al., 2016). *Glyma03g32900* affects Na^+^ and Cl^−^ accumulation in soybean shoot under salt stress (Do et al., 2016; Liu et al., 2016). Recently, the association of *Glyma03g32900* with Na^+^ and Cl^−^ accumulation in leaf was verified by genetic mapping of QTLs related to leaf Na^+^ and Cl^−^ contents (Do et al., 2018). In this study, using a population derived from the salt-sensitive cultivar Zhonghuang39 and the salt-tolerant wild soybean NY36-87, we mapped a dominant tolerance gene on Chr. 03 to a 98-kb interval, which included *GmSALT3* (*Glyma03g32900*; Figure 2A). This indicated that the tolerance gene *Glyma03g32900* is conserved in salt-tolerant germplasm.

The availability of high-throughput genome resequencing and SNP arrays promoted the identification of salt tolerance-related genes from diverse soybean germplasm (Zeng et al., 2017a; Jin et al., 2021). Two tolerant QTLs were mapped on Chr. 13 and Chr. 15, with *Glyma.13g161800* and *Glyma.15g091600* proposed as the candidates (Zeng et al., 2017b). A novel dominant locus related to leaf sodium content was mapped on Chr. 13, and the functional allele was from salt-sensitive parent Williams 82 (Do et al., 2018). These results showed that novel loci underlie salt tolerance in diverse soybean germplasm, even in salt-sensitive accessions. A similar result was reported related to soybean seed weight; the *PP2C-1* allele from the small-seeded wild soybean, but not from big-seeded cultivars, is responsible for seed weight promotion (Lu et al., 2017). In association analysis of *Glyma03g32900* functional markers with salt tolerance, several soybean accessions that may carry novel salt tolerance loci other than *Glyma03g32900* were found (Guan et al., 2014b; Patil et al., 2016; Lee et al., 2018). Peking is one such accession that is salt sensitive but contains the tolerant allele of *GmSALT3*. In the F_2_ population of Peking×NY36-87, the segregation of salt-tolerant and salt-sensitive genotypes fit a simple Mendelian ratio, suggesting that a dominant gene confers the salt tolerance in NY36-87. A novel salt tolerance-related locus on Chr. 18, which we called *GmSALT18*, was mapped to a 241-kb region (Figure 3B). There are 29 gene annotations based on the assembly of Williams 82 (Glyma.Wm82.a2.v1).^2^ Three genes, including *Glyma.18g026200* and *Glyma.18g026500*, which may relate to salt stress (Gong et al., 2001; Bouchabke-Coussa et al., 2008), and a predicted K^+^/H^+^-antiporter, *Glyma.18g027900*, were located in this region. In addition, potential genomic structure variations between *Glyma.18g026700* and *Glyma.18g026900* were observed in assemblies of soybean ZH13, wild soybean W05 and PI 483463 (Supplementary Figure 2). Given the facts that variation in *GmSALT18* of Peking may be a minor allele, and possible genomic structure variation, further study of fine mapping and genome sequencing of Peking and NY36-87 could be useful for successful identification of the candidate gene.

The discrepancy between genotype and phenotype for a particular gene, such as *GmSALT3*, may be due to the phenotype sorting method making it difficult to distinguish between moderately tolerant or sensitive germplasm (Patil et al., 2016). In this study, we classified salt tolerance as a binary (normal growth of plants=salt tolerant, plant death=salt sensitive) rather than rating salt tolerance on a 1–5 scale (Figure 1); this provided a more exact phenotype for gene mapping. Using a RIL population of Kefeng No. 1 (salt sensitive)×Nannong1138-2 (salt tolerant), a major QTL (qtrG.1) for salt tolerance ratings was previously located on Chr. 18 flanked by Sat_164 and Sat_358 (Chen et al., 2008), which is 9Mb from *GmSALT18*. This suggests that *GmSALT18* may represent a novel salt tolerance locus in wild soybean. Considering that the expression patterns of *GmSALT3* in Peking and NY36-87 were similar (Figure 5B), it is unlikely that *GmSALT18* plays a role in regulation of *GmSALT3*. Peking accumulated more Na^+^ and K^+^ in leaves compared to salt tolerant germplasm (Figure 4), as was observed in salt-sensitive cultivars 85–140 and NIL-S, which contain the sensitive *GmSALT3* allele (Guan et al., 2014b; Qu et al., 2021). The mechanism underlying Na^+^ accumulation in Peking thus requires further study.

In summary, through mapping of salt tolerance genes using two F_2:3_ populations, we identified the salt tolerance gene *GmSALT3* in the population Zhonghuang39×NY36-87. In the population Peking×NY36-87, a novel locus, *GmSALT18*, was found to be responsible for the difference in salinity tolerance between Peking and NY36-87. Further research is needed to clone the corresponding gene underlying *GmSALT18* and demonstrate the genetic relationship between *GmSALT3* and *GmSALT18*.

## Data Availability Statement

The original contributions presented in the study are included in the article/Supplementary Material, further inquiries can be directed to the corresponding authors.

## Author Contributions

RG, RC, and LQ designed the research. XG, JJ, and LY performed the salt tolerance phenotype evaluation. LY and XG performed salt stress and gene expression. XG performed fine mapping of the salt tolerance genes and dada analysis. XG, RG, and LQ wrote the manuscript. All authors contributed to the article and approved the submitted version.

## Funding

This research was funded by the Natural Science Foundation of China (31830066) and Central Public-interest Scientific Institution Basal Research Fund (S2021ZD02).

## Conflict of Interest

The authors declare that the research was conducted in the absence of any commercial or financial relationships that could be construed as a potential conflict of interest.

## Publisher’s Note

All claims expressed in this article are solely those of the authors and do not necessarily represent those of their affiliated organizations, or those of the publisher, the editors and the reviewers. Any product that may be evaluated in this article, or claim that may be made by its manufacturer, is not guaranteed or endorsed by the publisher.

## Footnotes

^1^ http://www.isbreeding.net

^2^ https://www.soybase.org

## Supplementary Material

Supplementary Figure 1 Distribution of 543 simple sequence repeat (SSR) markers used for polymorphic markers selection in bulked segregant analysis (BSA) analysis of F_2_ population derived from a cross of Peking and NY36-87.

Supplementary Figure 2 Physical position of the potential genomic structure variation (between *Glyma.18g026700* and *Glyma.18g026900*) in different assemblies of soybean accessions on Chr. 18. Box indicates where the variations locate.
