# Supplementary figures and images for: Identification of a Novel Salt Tolerance-Related Locus in Wild Soybean (Glycine soja Sieb. & Zucc.)
Source: Front Plant Sci. 2021 Nov 18;12:791175. doi: 10.3389/fpls.2021.791175 (PMC8637416; doi:10.3389/fpls.2021.791175)

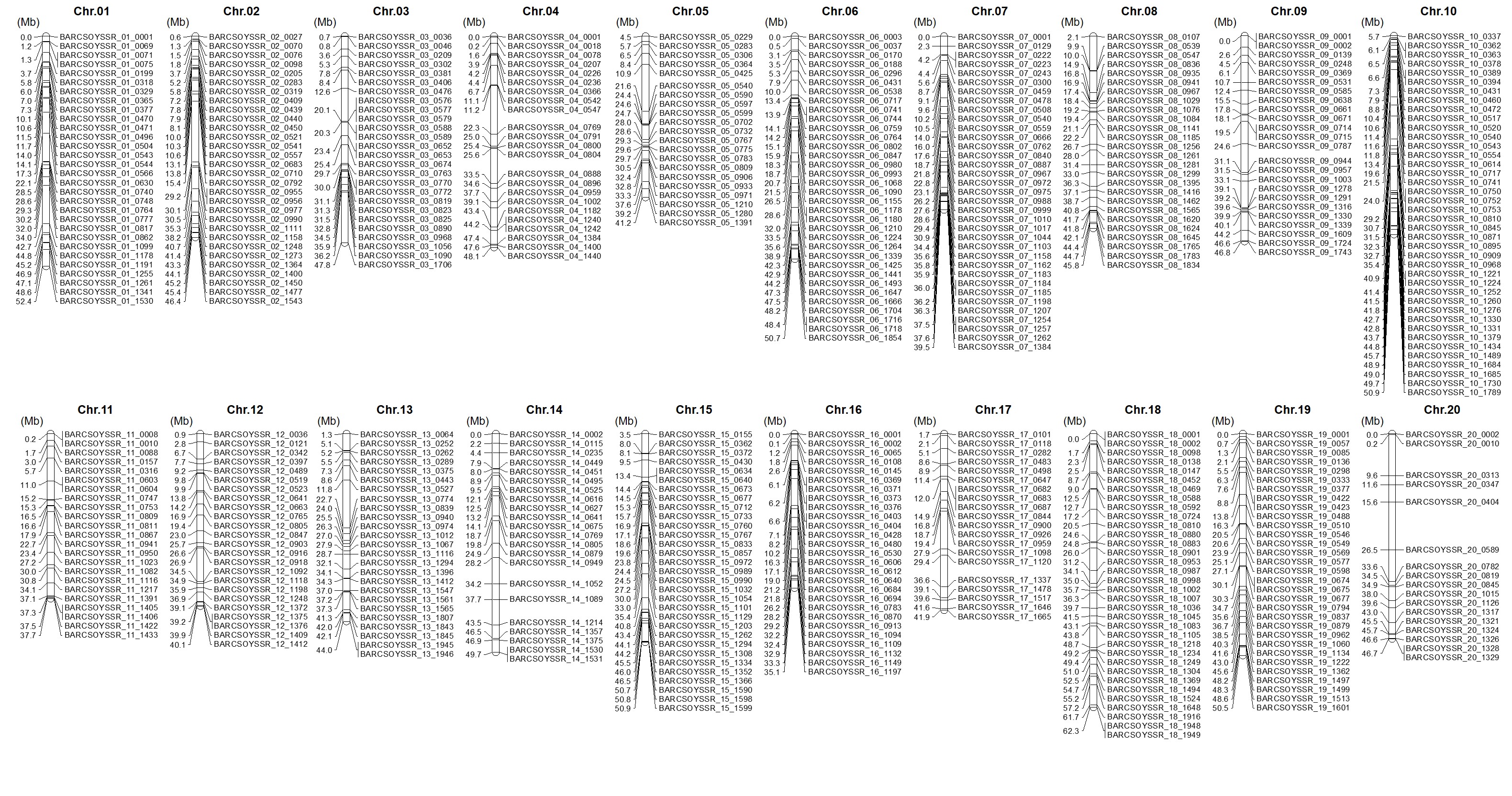

Supplement: Supplementary Figure 1 — Distribution of 543 simple sequence repeat (SSR) markers used for polymorphic markers selection in bulked segregant analysis (BSA) analysis of F2 population derived from a cross of Peking and NY36-87. [file Image_1.JPEG]

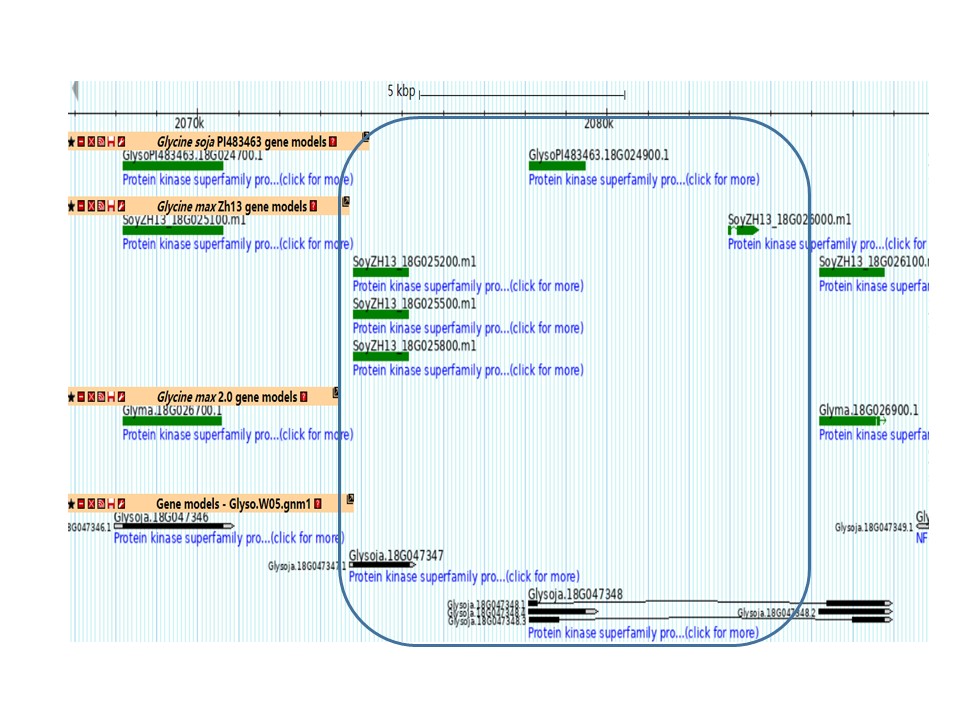

Supplement: Supplementary Figure 2 — Physical position of the potential genomic structure variation (between Glyma.18g026700 and Glyma.18g026900) in different assemblies of soybean accessions on Chr. 18. Box indicates where the variations locate. [file Image_2.JPEG]
