# Supplementary material for: Identification of a Novel Salt Tolerance-Related Locus in Wild Soybean (Glycine soja Sieb. & Zucc.)
Source: Front Plant Sci. 2021 Nov 18;12:791175. doi: 10.3389/fpls.2021.791175 (PMC8637416; doi:10.3389/fpls.2021.791175)
Supplement: Supplementary file 3 [file Table_1.DOCX]

| **SUPPLEMENTARY TABLE 1 \| Primers used in gene mapping and qRT-PCR** | | | | | |
| --- | --- | --- | --- | --- | --- |
| **Primer name** | **Marker type** | **Forward(5'-3')** | **Reverse(5'-3')** | **Position** | **Purpose** |
| BARCSOYSSR_03_1329 | SSR | TCAAGAACAAACTGCACATTCC | CGTGTACTACCCTGAAGTGGC | 38,425,595 | Primers for mapping in Chr.03 |
| BARCSOYSSR_03_1332 | SSR | ACAAAATTTGACAGTGAAGTTGATA | GCGACATTTCTTAATGGGGA | 38,431,824 |  |
| BARCSOYSSR_03_1336 | SSR | GGCAGTGCTGTGAAAACTCA | AGAACCCATGACAAAAACTTGA | 38,548,422 |  |
| H2-Ins | InDel | GCGGGAGTAATGTTATCGG | CTATTCTCATAAGAGTCTA | 38,615,737 |  |
|  |  |  | GTCGTATCTTGGGAGAGGAG |  |  |
| BARCSOYSSR_03_1342 | SSR | GTCAACAGAACCGAGACCGT | ATTCACATCCTGAGCTTGGC | 38,649,535 |  |
| BARCSOYSSR_03_1349 | SSR | CGTCCTCACTCACTCACCAA | GAACCCGAATTTCTCGAACA | 38,858,688 |  |
| BARCSOYSSR_03_1361 | SSR | CCAGAGGAACAACCTTGGAA | GTCCCTCTCTGCATGACCAA | 39,182,032 |  |
| BARCSOYSSR_03_1380 | SSR | GCATCCAAGGCTCTCAAGAC | ATACACCTTGGCCACTTTGC | 39,632,918 |  |
| BARCSOYSSR_18_0001 | SSR | CAGTTGAATGCCATTATTTTTCAG | CAGGGCTTTTTGTATCAGCA | 25,736 | Primers for mapping in Chr.18 |
| BARCSOYSSR_18_0067 | SSR | TGAATTTCACCGTGTTTATGATTT | GCAAAATGATTCCCCTTGTT | 1,372,390 |  |
| BARCSOYSSR_18_0082 | SSR | TGGCCTTAATACGTGTCACAA | TGGTTTCTATTTACGGGTACCAA | 1,509,516 |  |
| BARCSOYSSR_18_0098 | SSR | TTTTGAGAGATAGACAAATATTCCC | GGGTGTCTTTTTGGTTGAGG | 1,691,447 |  |
| BARCSOYSSR_18_0102 | SSR | GCGCCTTCAAATTGGCGTCTT | GCGCCTTAAATAAAACCCGAAACT | 1,736,741 |  |
| BARCSOYSSR_18_0103 | SSR | TCATTTAAGAAATCTTTGACTACAAAA | CCCACTTCAATTAACTCATGC | 1,755,117 |  |
| BARCSOYSSR_18_0107 | SSR | GGATGTTGGATGCACATGAA | GGTCAACTTTTGTTGATACATCG | 1,851,155 |  |
| BARCSOYSSR_18_0112 | SSR | CCAGAGGAAACAGGGAGTCA | CTCAACAAGGCAGGCATGTA | 1,947,294 |  |
| BARCSOYSSR_18_0120 | SSR | GTTGCTTGAGTGCTCGACTG | GTTTTGGCACGAGCTTTCTC | 2,092,590 |  |
| BARCSOYSSR_18_0121 | SSR | TTGTGTGGGGTTGATTTGAA | CAGGAAGTTTGTATTGAGGCAA | 2,100,691 |  |
| BARCSOYSSR_18_0122 | SSR | TGAGTGTCGTATATGATATTTGCTTG | TGATTTGCAACAGACGAACAG | 2,111,031 |  |
| BARCSOYSSR_18_0123 | SSR | CAGCATGCTCTTTTCTCCCT | GTCGCACCAGCATTTGTTAT | 2,137,025 |  |
| BARCSOYSSR_18_0125 | SSR | TTCCCACGTGTCAACTTCTG | TTGTCACCTCCATCATACACA | 2,186,042 |  |
| (Continued on the next page) | | | | | |
| (Continued from previous page) | | | | | |
| **Primer name** | **Marker type** | **Forward(5'-3')** | **Reverse(5'-3')** | **Position^*^** | **Purpose** |
| BARCSOYSSR_18_0146 | SSR | CCGAGGTACACAAGAAGCAA | CCACACCCCATGAAACAAAT | 2,531,185 | Primers for mapping in Chr.18 |
| BARCSOYSSR_18_0155 | SSR | CACATCACTCGGGAGATTTTT | GGGATGAGAGGGTGGAGATT | 2,644,060 |  |
| *GmUKN1* |  | TGGTGCTGCCGCTATTTACTG | GGTGGAAGGAACTGCTAACAATC |  | qRT-PCR |
| *q-SALT3* |  | TCCTTGACGCTTGGAGTGTT | CGGTTGATGAAGGGAAAAC |  | qRT-PCR |
